# Prevalence of *Helicobacter pylori* Infection and Efficacy of Bismuth Quadruple and Levofloxacin Triple Eradication Therapies: A Retrospective Analysis

**DOI:** 10.3390/life14070885

**Published:** 2024-07-17

**Authors:** Patricia Serena, Alexandru Popa, Renata Bende, Bogdan Miutescu, Ruxandra Mare, Andreea Borlea, Giovanni Aragona, Andrei Lucian Groza, Luca Serena, Alina Popescu, Ioan Sporea, Roxana Sirli

**Affiliations:** 1 Division of Gastroenterology and Hepatology, Department of Internal Medicine II, Centre for Advanced Research in Gastroenterology and Hepatology, “Victor Babes” University of Medicine and Pharmacy, E. Murgu Square, Nr. 2, 300041 Timisoara, Romania; patricia.lupulescu@umft.ro (P.S.); renatabende29@gmail.com (R.B.); miutescu.bogdan@umft.ro (B.M.); mare.ruxandra@umft.ro (R.M.); popescu.alina@umft.ro (A.P.); isporea@umft.ro (I.S.); sirli.roxana@umft.ro (R.S.); 2 Division of Endocrinology, Department of Internal Medicine II, “Victor Babes” University of Medicine and Pharmacy, E. Murgu Square, Nr. 2, 300041 Timisoara, Romania; borlea.andreea@umft.ro; 3 Gastroenterology and Hepatology Unit, Guglielmo da Saliceto Hospital, 29121 Piacenza, Italy; g.aragona@ausl.pc.it; 4 3rd Department of Internal Medicine, Iuliu Hatieganu University of Medicine and Pharmacy, 400012 Cluj-Napoca, Romania; groza.andrei@umfcluj.ro; 5 Anaesthesia and Intensive Care Department, Guglielmo da Saliceto Hospital, 29121 Piacenza, Italy; dr.serenaluca@gmail.com

**Keywords:** *Helicobacter pylori*, eradication, prevalence, gastritis, gastroduodenal ulcers, gastric cancer, upper endoscopy

## Abstract

*Helicobacter pylori* (*H. pylori*) infection is a widespread global health issue and a primary cause of diseases like gastritis, gastroduodenal ulcers, and gastric cancer. This study examines the prevalence of *H. pylori* infection in patients undergoing upper endoscopy and assesses the efficacy of bismuth quadruple therapy (BQT) and levofloxacin triple therapy. A retrospective analysis of 507 gastroscopies was conducted, with indications including epigastric pain, heartburn, postprandial fullness, early satiation, and regurgitation. Rapid urease tests were performed, and endoscopic findings documented. Two treatment regimens were used: BQT as the first-line therapy and levofloxacin triple therapy as the second-line. Of the 507 patients, 68.8% were infected with *H. pylori*. Gastric ulcer patients had significantly higher *H. pylori* prevalence compared to those with small polyps, Barrett’s esophagus, or normal endoscopy. Among the 310 patients who participated in follow-up interviews, 11.9% did not initiate therapy and 5.1% discontinued due to intolerance. The overall eradication rate was 88.6%, with BQT showing a higher eradication rate (89.4%) compared to levofloxacin triple therapy (83.8%). The study highlights the high prevalence of *H. pylori* among patients with gastrointestinal symptoms and the effectiveness of BQT as a first-line treatment.

## 1. Introduction

*Helicobacter pylori* (*H. pylori*) ranks among the leading causes of chronic bacterial infections and is classified as a class I carcinogen by the World Health Organization [1]. The prevalence of *H. pylori* infection varies greatly depending on age, associated diseases, geographic regions, race/ethnicity, socioeconomic status, and hygienic conditions [2,3,4,5]. Epidemiological studies have shown that the global prevalence of *H. pylori* has declined in the last three decades, from 52.6% to 43.9 in adults, but was still as high as 35.1% in children and adolescents [6,7,8,9,10]. According to the latest study conducted in Romania, the prevalence of *H. pylori* infection is reported to be 40% [11].

Patients with *H. pylori* infection can present with dyspeptic symptoms, but many individuals remain asymptomatic. If not eradicated, *H. pylori* can eventually lead to gastritis, peptic ulcer disease, and gastric mucosa-associated lymphoid tissue lymphoma. It has also been shown that *H. pylori* infection is present in 95% of cases of gastric cancer [12]. Lowering the prevalence of *H. pylori* infection could potentially decrease mortality from gastric cancer. Hence, effectively eradicating *H. pylori* infection in clinical settings is crucial for disease prevention efforts [13].

Routine susceptibility testing prior to prescribing first-line *H. pylori* treatment can help assess antibiotic resistance and improve treatment effectiveness. Without susceptibility testing, clinicians should consider local antibiotic resistance rates. In areas where data is lacking, assuming high clarithromycin resistance is advisable [12]. Bismuth quadruple therapy (BQT), which consists of a proton pump inhibitor (PPI), bismuth, tetracycline, and metronidazole, is currently the recommended first-line empiric therapy for *H. pylori* infection. Regarding second-line treatment after first-line eradication therapy failure, the Maastricht VI/Florence 2022 Consensus recommends either a bismuth-containing quadruple therapy, a fluoroquinolone-containing quadruple (or triple) therapy, or a PPI–amoxicillin high-dose dual therapy [12]. Consensus conferences on the eradication of *H. pylori* infection recommend using treatments that achieve a minimal cure rate of 90%, as none of the available therapies to date reach 100% effectiveness.

Several risk factors can contribute to the failure of *H. pylori* eradication. Patient-related factors include poor compliance with the treatment regimen, smoking, alcohol consumption, and obesity [14,15,16]. Also, non-compliance with pharmacological treatment, also known as therapeutic non-compliance, is a prevalent and significant issue in clinical practice. Compliance rates for *H. pylori* eradication treatment vary based on factors such as the country, age group (pediatrics versus adults), type of study (real-life versus clinical trial), and the duration of therapy [17,18,19,20]. Factors associated with low compliance include the indication of treatment for functional dyspepsia, rescue treatment, longer prescriptions, adverse events, and receiving sequential or concomitant treatment. Proper compliance is the variable most closely associated with successful eradication [21]. 

Every unsuccessful attempt at eradication can contribute to the development of antibiotic resistance, complicating future treatments and exacerbating regional antibiotic resistance levels [22].

The aim of our study was to determine the prevalence of *H. pylori* infection in patients presenting with gastrointestinal symptoms in a tertiary gastroenterology department and to assess the efficacy of two different eradication regimen—a 14-day BQT regimen in naïve patients with *H. pylori* infection and a 14-day levofloxacin triple therapy as a rescue treatment. 

## 2. Materials and Methods

### 2.1. Research Design and Ethical Considerations

A detailed retrospective analysis was conducted, examining the medical records of a cohort of 556 individuals who had undergone gastroscopy between January and December 2022. The research was carried out in a tertiary healthcare facility situated in western Romania. Digital and paper records were used to identify the main complaints, demographic data, and additional clinical data. This research adhered to the ethical principles outlined in the 1975 Declaration of Helsinki and received approval from the internal review board.

### 2.2. Participant Selection and Sample Collection

The study enrolled subjects aged 18 years and older who had undergone both gastroscopy and rapid urease testing (RUT). The indications for gastroscopy covered a range of upper gastrointestinal symptoms and diagnostic needs, including persistent or recurrent manifestations such as epigastric pain, heartburn, postprandial fullness, early satiation, and gastroesophageal reflux disease (GERD). Gastroscopy was also used to investigate anemia due to gastrointestinal bleeding, monitor pre-existing gastrointestinal conditions, assess unexplained weight loss, and evaluate suspected gastrointestinal hemorrhage.

Specific exclusion criteria were applied, excluding individuals under the age of 18 and patients with decompensating diseases (hepatic, cardiac, pulmonary, or renal), severe mental health disorders, pregnancy, breastfeeding, previously known malignancy, and gastric cancer. These criteria ensured a homogeneous and clinically relevant patient population for the study’s analyses. During the endoscopic procedures, all patients were sedated with benzodiazepines (Midazolam), fentanyl and propofol, and gastroscopies were performed using Olympus Evis EXERA III (CV-190) and GIF-HQ190 endoscopes (Olympus corp., Tokyo, Japan). Biopsies were taken from the antrum for RUT to determine the presence or absence of *H. pylori*, using the AMA RUT Pro test (AMA-Med Oy, Mikkeli, Finland). The AMA RUT Pro test works by changing the color of the reactive element after the biopsy specimen is placed on its surface. If urease activity was present in the biopsy specimen, a red or magenta spot appeared on the back of the reactive surface. The test provides rapid results, with color change occurring in only 5 min, and has high accuracy (99.25% sensitivity and 98.76% specificity). Mucosal samples from the gastric antrum were collected for subsequent diagnostic testing. Simultaneously, endoscopic findings were documented, comprising a broad range of observations including gastritis/duodenitis, hiatal hernia, esophagitis, gastric ulcer, duodenal ulcer, small polyps, Barrett’s esophagus, and other pathological findings.

### 2.3. Treatment Strategies for H. pylori-Positive Patients

Two different eradication regimens were used based on the existence of previous *H. pylori* infection. A subset of patients within the cohort reported prior *H. pylori* infection and subsequent treatments, although they couldn’t recall the specific therapeutic interventions administered. In these cases, the patients followed the second-line treatment with levofloxacin triple therapy. Alternatively, the other subset of patients did not recall previous diagnoses of *H. pylori* infection, prompting the utilization of a first-line treatment approach employing bismuth quadruple therapy. This stratified approach aimed to optimize therapeutic efficacy by tailoring treatment regimens to individual patient histories and maximizing the likelihood of successful eradication of the *H. pylori* infection.

Levofloxacin triple therapy included: esomeprazole 40 mg orally, twice daily for 14 days; amoxicillin 1 g orally, twice daily for 14 days; and levofloxacin 500 mg orally twice daily for 14 days. The BQT regimen consisted of: esomeprazole 40 mg orally, twice daily for 14 days; metronidazole 500 mg orally, twice daily for 14 days. tetracycline 500 mg, four times daily for 14 days; and bismuth subsalicylate 120 mg, four times daily for 14 days.

### 2.4. Monitoring and Follow-Up

Patients were contacted via phone interviews to assess treatment compliance, including adherence to the prescribed therapeutic regimen or premature discontinuation. Additionally, the interviews served to determine whether patients underwent fecal antigen testing four weeks after treatment completion to confirm the eradication status of *H. pylori* infection. This methodological approach facilitated the collection of pertinent clinical data regarding treatment adherence and infection eradication outcomes, thereby contributing to the comprehensive evaluation of therapeutic efficacy.

## 3. Results

### 3.1. Baseline Characteristics

Five hundred and seven consecutive adult subjects, with ages ranging from 18 to 88 years old, and a mean age of 57.15 ± 13.89 years, including 191 men (37.7%) and 316 women (62.3%), all of whom underwent upper digestive endoscopy with RUT for *H. pylori*, were included. The baseline characteristics, demographic data, laboratory parameters, and endoscopic findings are presented in Table 1.

### 3.2. H. pylori Prevalence

RUT for *H. pylori* was performed by obtaining gastric antrum mucosa samples in 507 subjects, and *H. pylori* infection was detected in 349 patients (68.8%). The prevalence of *H. pylori*, according to endoscopic findings, is summarized in Table 2. The prevalence of *H. pylori* was significantly higher in subjects with gastric ulcer compared to those with small polyps (*p* = 0.0033), Barrett’s esophagus (*p* = 0.0020), other lesions or normal endoscopy (*p* < 0.0001). No significant differences were found between subjects with gastritis/duodenitis and gastric ulcer (*p* = 0.4663), duodenal ulcer (*p* = 0.5144) or esophagitis (*p* = 0.5004) (Table 2).

**Figure 1:**
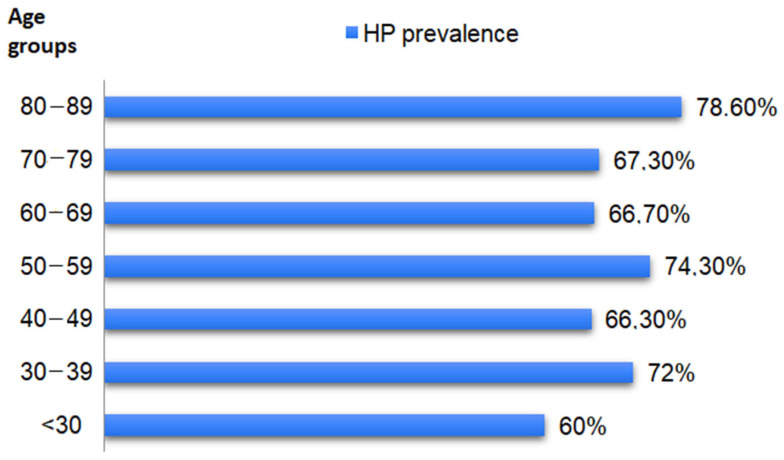
The distribution of positive cases according to age subgroups.

In a more detailed analysis, according to age subgroups, the highest prevalence of HP infection was in the 80–88 age subgroup (78.6%—11/14), without statistically significant differences between groups. The distribution of positive cases, by age group, is illustrated in Figure 1.

### 3.3. Follow-Up and Treatment in H. pylori-Positive Subjects

**Figure 2:**
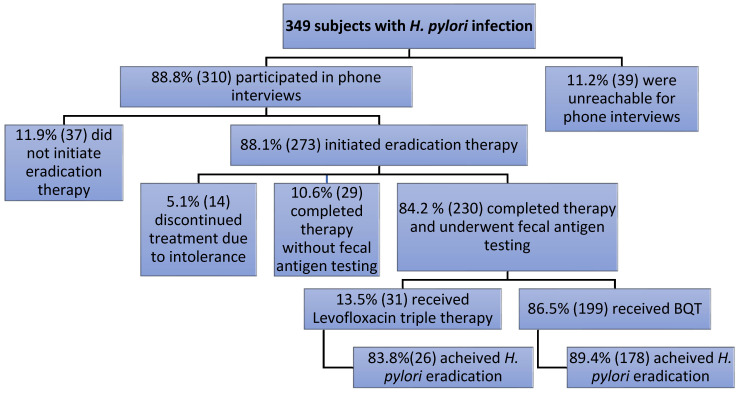
Systemic representation of the distribution of patients.

In our study, 349 subjects with *H. pylori* infection were initially identified. Of these, 11.2% (39/349) were unreachable for phone interviews. The remaining 310 patients participated in phone interviews. A systematic representation of the distribution of patients is summarized in Figure 2. 

Among the 310 interviewed subjects, 11.9% (37/310) did not initiate eradication therapy. Of the 273 patients who initiated therapy, 5.12% (14/273) discontinued treatment due to intolerance, while 10.62% (29/273) completed therapy without undergoing fecal antigen testing. 

A total of 84.24% (230/273) completed therapy and underwent fecal antigen testing. Of the 230 who completed therapy and underwent fecal antigen testing, 199/230 received BQT, while 31/230 received levofloxacin triple therapy.

Regarding the eradication of *H. pylori* infection, the overall eradication rate was 88.6% (204/230), higher for subjects treated with BQT (178/199), compared to those treated with levofloxacin triple therapy (26/31) (89.4% vs. 83.8%) but without reaching statistical significance (*p* = 0.5420)

The reported adverse events leading to discontinuation included gastrointestinal symptoms such as abdominal discomfort, nausea, and metallic taste; notably, all these patients were on BQT therapy, with none of the patients on levofloxacin reporting adverse events.

## 4. Discussion

### 4.1. Literature Findings

*H. pylori* infection is a common global health concern, affecting over half of the world’s population. Its prevalence varies widely by region, influenced by factors such as socioeconomic conditions and healthcare access [2]. The prevalence of *H. pylori* infection in our study cohort was 68.8%. This is significantly higher than the reported national prevalence of 40% in Romania, underscoring the substantial prevalence of this pathogen among patients undergoing gastroscopy. This disparity could be attributed to the selection criteria of our study population, which focused on patients presenting with gastrointestinal symptoms, who are more likely to have underlying conditions associated with *H. pylori* infection, thereby increasing the detected prevalence rate.

In 2021, Ribaldone et al. conducted a retrospective study in Italy and observed a notably lower prevalence of 16.8% [23]. Several factors could explain this difference. The Italian study included a broader population with varying endoscopic findings: 16.1% of patients had normal endoscopic findings, while only 38.7% had gastritis or duodenitis. In contrast, our study had 82.6% of patients with gastritis and only 2% with normal endoscopic findings. The higher prevalence of gastritis in our cohort likely contributed to the higher *H. pylori* detection rate, as *H. pylori* is a well-known cause of gastritis. Moreover, the regional differences between Romania and Italy, including variations in socioeconomic factors and healthcare access, may also influence the prevalence rates. The Italian study further highlighted that the prevalence of *H. pylori* was significantly higher among patients born in Eastern Europe (32.5%) compared to those born in Italy. This finding aligns with our study conducted in Romania, where the prevalence of *H. pylori* infection remains high. 

Similarly, another study conducted in Turkey aimed to investigate the prevalence of *H. pylori* infection in patients undergoing endoscopy, including 2065 patients from January 2017 to January 2021. *H. pylori* positivity was detected in 52.15% of patients [24]. In South America, a 2020 study in Cali, Colombia, examined 613 patients undergoing upper endoscopy, primarily for dyspepsia, and found an *H. pylori* prevalence of 38.5% [25]. The varying prevalences of *H. pylori* infection across different regions underscore the influence of geographical and demographic factors on disease prevalence. These differences highlight the importance of considering regional variations when interpreting prevalence data and designing targeted intervention strategies. 

Despite 30 years of therapeutic experience worldwide, the ideal eradication regimens for H. pylori remain unclear. Therapy for *H. pylori* is also impacted by the global increase in antimicrobial resistance, which threatens the continued usefulness of currently available antimicrobials [26]. A study conducted in Romania recently examined the antimicrobial resistance patterns of *H. pylori* strains in Northwestern and Central Romania. Primary resistance against clarithromycin, fluoroquinolones, and metronidazole was found in 16.7%, 11.1%, and 13.3% of strains, respectively [27]. According to the Maastricht VI/Florence 2022 Consensus, if individual susceptibility testing is not available, the first-line recommended treatment in areas of high (>15%) or unknown clarithromycin resistance is bismuth quadruple therapy. Given the 16.7% resistance rate to clarithromycin in our study, BQT is advisable in our area [12]. 

In this study we assessed the efficacy of two different eradication regimens—a 14-day BQT regimen in naïve patients with *H. pylori* infection versus a 14-day levofloxacin triple therapy as a rescue treatment. We found that both therapies achieved the overall eradication rate of 88.6% in a real-world setting. The eradication rate for *H. pylori* infection using BQT as a first-line treatment was 89.4%, demonstrating a high level of efficacy. This aligns with other studies supporting the effectiveness of BQT in eradicating *H. pylori*. For comparison, a recent study by Losurdo et al. in southern Italy reported a 100% success rate for BQT [28]. Although this rate is higher than what we observed, it is important to note that their study had a much smaller sample size, with only 48 patients following this therapy. In contrast, our study included a significantly larger cohort of 230 patients, providing a more robust evaluation of BQT’s effectiveness in a diverse population. 

Additionally, the Hp-EuReg analyzed the effectiveness and safety of a 10-day single-capsule BQT regimen in real-world settings across several European countries, including Spain, Italy, and Portugal, reporting an efficacy of 94.6% for first-line therapy [29]. Compared to these findings, our study’s eradication rate of 89.4% is slightly lower but still represents satisfactory results within the context of a larger and potentially more varied patient population.

Overall, our study confirms that BQT is a highly effective first-line treatment for *H. pylori* infection. The slight variations in eradication rates between different studies could be attributed to differences in sample size, patient demographics, adherence rates, and regional variations in antibiotic resistance. Nonetheless, our findings reinforce the utility of BQT in clinical practice, offering a reliable option for *H. pylori* eradication. 

Regarding the second-line treatment after first-line eradication therapy failure, The Maastricht Consensus recommends either a bismuth-containing quadruple therapy, a fluoroquinolone-containing quadruple (or triple) therapy, or a PPI-amoxicillin high-dose dual therapy [12]. In our study, the patients followed levofloxacin triple therapy as a second-line treatment and the eradication rate for *H. pylori* infection was 83.8%. This result, while substantial, highlights the ongoing challenge of achieving optimal eradication rates with empirical rescue therapy following initial treatment failure. Recent studies have evaluated combining a PPI–amoxicillin–levofloxacin triple therapy with bismuth to create a quadruple regimen, yielding encouraging results. One study by Gisbert et al., focusing on patients with one previous eradication failure, achieved an eradication rate of 90%, which is promising for empirical use [30]. The Hp-EuReg found that optimal eradication rates (≥90%) for second-line treatment were obtained with bismuth-containing quadruple therapy, with or without levofloxacin, but not with levofloxacin-based triple therapy alone [29]. This underscores the importance of bismuth-containing quadruple therapy as a pivotal second-line option for *H. pylori* eradication, particularly in regions with high quinolone resistance.

Given our study’s eradication rate of 83.8% for levofloxacin triple therapy, it is clear that while this regimen can be effective, it may not consistently achieve optimal eradication rates. More extensive studies with larger patient cohorts are needed to better assess the efficacy and safety of levofloxacin-based regimens. Additionally, exploring the combination of levofloxacin with bismuth in a quadruple therapy format could potentially enhance eradication rates and should be considered in future research.

Furthermore, our findings underscore the challenges associated with treatment adherence and intolerance. A substantial portion of patients either did not start or discontinued therapy. Among the 310 interviewed subjects, 11.9% (37/310) did not initiate eradication therapy. Of the 273 patients who initiated therapy, 5.12% (14/273) discontinued treatment due to adverse events. The adverse events encountered included gastrointestinal symptoms such as abdominal discomfort, nausea, and metallic taste; notably, all these patients were on BQT therapy, with none of the patients on levofloxacin reporting adverse events.

The fact that a significant number of patients (37) did not initiate therapy highlights the critical importance of patient education. Physicians must effectively communicate the potential temporary and mostly harmless side effects to patients to ensure they understand the importance of completing the treatment. Moreover, the discontinuation rate due to adverse events, at 5.12%, is high. The Hp-EuReg recently released a study examining the significance of compliance in the treatment of *H. pylori* eradication. Their research revealed that *H. pylori* eradication treatment typically demonstrates high adherence rates in real-world clinical settings, with a remarkably low non-compliance rate of just 1.7%. Factors contributing to lower compliance rates included treatment indication for functional dyspepsia, the need for rescue treatment, extended prescription durations, adverse events, and the administration of sequential or concomitant therapies [21]. Compared to the Hp-EuReg findings, our study had a higher non-compliance rate. Once again, this underscores the necessity of patient education to mitigate concerns about side effects and emphasize the importance of adhering to the treatment regimen.

The published rates of non-compliance exhibit considerable variability, influenced by a multitude of factors. These rates, as observed in clinical practice studies such as ours, span a wide spectrum, ranging from 17.1% in the investigation by Li et al. [18] to 5% in the research conducted by Romano et al. [31]. Factors correlated with higher compliance levels include the absence of adverse events, a treatment duration of 7 days, elevated doses of PPI, the indication for peptic ulcer, lack of prior treatment experience, and male gender. Adverse events emerge as a predominant cause of non-compliance with treatment. For instance, in the same study we mentioned above, Li et al. noted that 6.1% of patients (18 out of 293) discontinued treatment due to adverse events [18]. Similarly, a Spanish study reported that 7 out of 8 instances of discontinuation among non-compliant patients were attributable to adverse events [32]. In our study, all discontinuations were attributed to adverse events.

These findings highlight the significant role that treatment tolerability plays in the successful eradication of *H. pylori*. Improving patient education about potential side effects and the importance of completing the therapy could enhance adherence. Furthermore, developing therapies with better tolerability profiles could potentially reduce discontinuation rates and improve overall treatment success.

### 4.2. Study Limitations and Future Perspectives

As a retrospective cohort study, the quality of the analyzed data might be lower due to the inherent limitations of using digitally created data from paper medical records, which are prone to human error. Additionally, the selection criteria focused on patients presenting with gastrointestinal symptoms, which may have led to a higher detected prevalence rate of *H. pylori* infection compared to the general population. Also, the study did not have comprehensive data on factors such as alcohol use, tobacco use, and previous antibiotic usage, which could influence treatment outcomes.

Despite these limitations, all patients eligible for the study were carefully screened, and the sample size was large.

Future researchers should aim to conduct prospective studies to validate the observed prevalence of *H. pylori* infection and assess the efficacy of eradication regimens in diverse populations. Prospective studies are particularly needed to test the antibiotic resistance to fluoroquinolones and clarithromycin, as rising resistance levels pose significant challenges to effective treatment. Addressing these factors will provide a more comprehensive analysis and improve the overall understanding of *H. pylori* eradication.

## 5. Conclusions

In conclusion, we observed a significantly higher prevalence of *H. pylori* infection (68.8%) among patients undergoing gastroscopy. The efficacy of BQT as a first-line treatment for *H. pylori* was reaffirmed, achieving an eradication rate of 89.4%. The levofloxacin triple therapy, used as a second-line treatment, achieved an eradication rate of 83.8%, indicating the need for further research to optimize rescue therapies, particularly in regions with high quinolone resistance. Adherence to treatment regimens and management of adverse events are critical factors influencing eradication success. Our study highlighted a notable discontinuation rate due to adverse events, emphasizing the need for improved patient education on completing therapy despite potential side effects.

Our study contributes valuable data on the prevalence and treatment efficacy of *H. pylori* in Romania, reinforcing the need for targeted intervention strategies and continuous evaluation of treatment protocols.

## Figures and Tables

**Table 1:** Patients’ characteristics.

Parameter	Subjects *n* = 507
Mean age (years)	57.15 ± 13.89
Age subgroups	
<30	3.9% (20/507)
30–39	8.5% (43/507)
40–49	16.4% (83/507)
50–59	20.7% (105/507)
60–69	28.4% (144/507)
70–79	19.3% (98/507)
80–88	2.8 (14/507)
Gender	
Males	191/507 (37.7%)
Females	316/507 (62.3%)
Mean BMI (kg/m^2^)	28.13 ± 5.59
Place of residence	
Urban	50.5% (256/507)
Rural	49.5% (251/507)
Endoscopic findings	
Gastritis/Duodenitis	82.6% (419/507)—342 poz
Hiatal hernia	38.7% (196/507)—144 poz
Esophagitis	10% (51/507)—39 poz
Gastric ulcer	2.8% (14/507)—13 poz
Duodenal ulcer	1% (5/507)—3 poz
Small polyps	2.6% (13/507)—4 poz
Barrett’s esophagus	2.8% (14/507)—4 poz
Other lesions	4% (20/507)—2 poz
Normal endoscopy	2% (10/507)—2 poz

**Table 2:** Prevalence of HP according to endoscopic findings.

No	Endoscopic Findings				Prevalence of HP	
I	Gastritis/Duodenitis				81.6% (342/419)	
II	Hiatal hernia				73.5% (144/196)	
III	Esophagitis				76.5% (39/51)	
IV	Gastric ulcer				92.9% (13/14)	
V	Duodenal ulcer				60% (3/5)	
VI	Small polyps				30.8% (4/13)	
VII	Barrett’s esophagus				28.6% (4/14)	
VIII	Other lesions				10% (2/20)	
IX	Normal endoscopy				20% (2/10)	
*p* value						
I/II	I/III	I/IV	I/V	I/VI	I/VII	I/VIII
0.0360	0.5004	0.4663	0.5144	<0.0001	<0.0001	<0.0001
*p* value						
I/IX	II/III	II/IV	II/V	II/VI	II/VII	II/VIII
<0.0001	0.7977	0.1948	0.8715	0.0030	0.0011	<0.0001
*p* value						
II/IX	III/IV	III/V	III/VI	III/VII	III/VIII	III/IX
0.0011	0.3259	0.7855	0.0051	0.0024	<0.0001	0.0019
*p* value						
IV/V	IV/VI	IV/VII	IV/VIII	IV/IX	V/VI	V/VII
0.3086	0.0033	0.0020	<0.0001	0.0013	0.5495	0.4781
*p* value						
V/VIII	V/IX	VI/VII	VI/VIII	VI/IX	VII/VIII	VII/IX
0.0608	0.3329	0.7668	0.2930	0.9158	0.3457	0.9987

## Data Availability

The data presented in this study are available on request from the corresponding author due to privacy concerns.
